# Illinois State Dental Board Affairs

**Published:** 1901-10-15

**Authors:** 


### ILLINOIS STATE DENTAL BOARD AFFAIRS.

In our last issue we stated that the task of investigating the bogus diploma mills and questionable methods of State boards was assigned to the Dental Protective Association by the National organizations while in session at Milwaukee, in August. Accordingly we secured the services of counsel and began the work of running down the various rumors and collecting evidence, so as to ascertain who were the guilty parties and to bring them to justice. We now give to our readers the following brief summary of what has been accomplished.

The first step was to lay the matter of the abuses existing in the State of Illinois before the proper authorities—the attorney general, and the State's attorney at Chicago, through which channel the investigation of the shortcomings of the Illinois State Board of Dental Examiners is being carried on. The next step was to secure reliable evidence of the misdemeanors which, it was claimed, had been committed.

We have already acknowledged the aid given us by the American Counsel at Munich, Germany, Hon. Jas. H. Worman,. who was early in the investigation and who labored diligently collecting evidence against the bogus diploma traffic in Germany. This matter has been published in a general way in most of the dental journals, under the head of "Report of the Foreign Relations' Committee of the National Association of Dental Faculties." We will on this account omit what must be already familiar to our readers and discuss more recent occurences relating to the subject in hand.

The prosecution having been begun in the courts of Chicago in the Gumpoldt matter, upon information furnished by Counsel Worman, and upon the advice of Hon. Charles S. Dineen, State's Attorney, a warrant was issued against J. H. Smyser, late secretary of the Illinois State Board of Dental Examiners, on the charge of forgery in issuing a license purporting to have been dated May 5, 1899, to one Emil Gumpoldt. Smyser was arrested and gave bail, but the prosecution was subsequently discontinued, because expert evidence tended to show that the license did not contain Smyser's handwriting, and also because the original license was in Germany and so was not immediately available for the case in hand.

The second complaint against Smyser was for uttering the Emil Gumpoldt license, upon which he was also arrested and gave bail. This proceeding was also discontinued owing to the absence of the original license.

The third charge was for the forgery of the authenticated proceedings of the Illinois State Board of Dental Examiners, whereby it is claimed that Smyser, on May 20, 1898, unlawfully issued to one Chas. A. Finley a license to practice dentistry in the State of Illinois. On this charge Smyser was arrested and gave bail, and a hearing was had before the Hon. Marcus A. Kavanaugh, Judge of the Superior Court, of Cook County, Illinois, sitting as a committing magistrate. On this hearing it appeared that C. A. Finley, to whom Smyser had issued the license May 20, 1898, had presented himself, along with a class of fifty-seven others, before the Board at a meeting held on March 21, 1896, for examination to procure a license to practice dentistry in the State; that of the fifty-eight applicants only eleven passed a successful examination, and that Finley was one of those who failed to pass and hence was not entitled to a license. It further appeared in evidence on said hearing that Finley had no diploma and was not a graduate of any reputable dental college. It also appeared in evidence that on or about May 17, 1898, more than two years after he had been examined by the State Board, he (Finley) presented an affidavit to Smyser in which he recited that he was 30 years old, a native of Indiana, a resident of Chicago, and that he had been a practitioner of dentistry ten years and had completed a two years full course of lectures and instructions in the N. W. C. The date of the license shows that after this affidavit was made by Finley and presented to Smyser, a license was issued to Finley. The reader will observe that Finley then obtained a license without a diploma and without any examination by the State Board other than that on March 21, 1896, in which he failed.

On March 21, 1896, at the time when Finley was examined and failed, Dr. L. L. Davis was secretary of the State Board of Dental Examiners, and the authentic minutes of that meeting were copied in the record book of the Board and testified to by Davis in court to be in his own handwriting. It was shown on the hearing that the record was kept by Davis, and we will quote therefrom: "The following named persons presented themselves for examination, the result being that the eleven first named obtained a permanent license to practice dentistry in Illinois."

Among those that failed C. A. Finley's was the nineteenth name. This name we detected had been changed from "C. A. Finley" to "C. A. Teteley." It was also discovered that the name of C. A. Finley had been written at the head of the list of successful candidates, and that the right-hand figure of the number 11 in the caption of the Board's proceedings, showing how many had passed a successful examination, had been changed from 1 to 2, making the result appear to be that 12 candidates had been successful instead of 11. It was also seen that the list of successful candidates had been further designated by numbers placed on the left-hand marginal side of their respective names. running from 1 to 12, and that figure 1 had been placed at the left of C. A. Finley's name, further indicating that he was at the head of the list.

Profs. Tolman and Drake gave expert testimony that in their opinion the name "C. A. Finley," as it appeard in the minutes written by Dr. Davis as the nineteenth unsuccessful candidate for license, had been changed to "C. A. Teteley," and that such change had been made by Dr. J. H. Smyser. That the name C. A. Finley, as written at the head of the list of successful candidates was in the handwriting of J. H. Smyser. That the right-hand figure 1 in the number 11 had been changed to a 2, that it had been made by J. H. Smyser, and that the numbering of the successful candidates was the work of J. H. Smyser. Dr. Smyser took the stand in his own defense, and denied making the change of "C. A. Finley" to "C. A. Teteley." He did not fully or clearly deny changing the 11 to 12, and before leaving the stand, on cross-examination by Assistant States Attorney Barnes, frankly admitted that he had written the name "C. A. Finley" at the head of the successful candidates. He offered no satisfactory explanation and we know of none to offer for him.

During Smyser's examination on this charge the State called C. A. Finley, who had made his affidavit that he had practiced dentistry for ten years prior to May 17, 1898. His attention being called to the affidavit, he testified in substance that he was in doubt whether the affidavit stated the facts intended when made. He was not clear in his own mind whether the affidavit as originally made stated that he had practiced for ten years or seven years; he thought it was seven years and that the affidavit must have been changed, but the handwriting experts were of the opinion that the affidavit was in Finley's handwriting and that any changes present had been made by him. Finley further admitted in his testimony that a considerable portion of this time, whatever the number of years, was devoted to study and acquiring a knowledge of dentistry and hence he could in no wise say of himself that he had been a dental practitioner for ten years prior to the date of his affidavit.

It was on this charge that Judge Kavanaugh, in holding Smyser on Sept. 9, 1901, to bail to await the action of the Grand Jury, said: "Since the adjournment on Saturday I have given this case careful consideration. The first charge against him (Smyser) in regard to the alteration of a record is, it seems to me, without defense—without serious defense. It appears unquestioned that the minutes of the Board were altered. It is hardly denied that Dr. Smyser made the alterations, so far as the names are concerned, and the testimony points conclusively to the fact that the man who made the alteration in regard to the name of Dr. Finley, and who wrote the numbers on the front of the book, must have been the man who made the alterations in the other letters and the other name.

"Now, the excuse given is not one that appeals to the judgement of anyone who has studied the circumstances. Here is a man who has presented himself to the State Board for examination and has failed. He remained content with that situation of affairs for almost two years. At the end of two years, the theory of the defense is, and the theory of Finley himself is, that he presented himself to Dr. Smyser and that Dr. Smyser, without consultation with any other member of the Board nor notice to them of what had been done, altered the face of the record upon the mere word of Dr. Davis, and here is a man licensed to practice dental surgery in this State where the record shows he is not entitled to do so. Now, there is a ring of probability in some portions of the recital of the defendant here, dependent not so much upon the evidence as upon that indefinable thing which comes to men who have spent many years in weighing evidence—the probability that some one else also is concerned in this. That some one else probably did introduce Mr. Finley to Dr. Smyser, and Dr. Davis upon the stand would not deny that it was probable that he himself had written that figure '2' upon the book, but even though somebody else were concerned in it, it is no excuse for this defendant. The alteration of that record is a forgery in view of the statue."

This charge in the Finley matter is awaiting the action of the Grand Jury of Cook County now in session.

The next offense for which complaint had been made against Smyser, and upon which he was arrested and held to bail, and upon which charge a hearing was had before Judge Kavanaugh, at the same time as in the Finley matter, was that of bribery. In this the arrest was made on the complaint of Oscar Cornelius Igney, a young man who alleged the purchase of a license from Smyser for the consideration of $400. It was testified by Igney in court upon the preliminary hearing to determine whether the crime of bribery had been committed or not, and whether or not there was probable proof of Smyser's guilt for which he could be held to await the action of the Grand Jury, that he (Igney) was a student in the Illinois School of Dentistry and had done considerable work and was employed in the laboratory of a dentist in this city named Haycock. That being desirous of obtaining admission to the dental profession at the earliest time possible he consulted Haycock prior to October last as to the advisability of his taking the State board examination, and was advised by Haycock that the easiest way to obtain a license was to purchase it, which could be done for a small sum of money, and Igney further testified that at Haycock's suggestion he placed in his hands $150.

A short time prior to the State board examination, which was held in October, 1900, Haycock returned to him a portion of the money, advising him that he would secure a license from the board. Igney took the examination but failed. He testified further that subsequently Haycock gave him a note and sent him to Smyser, which led to opening direct negotiations and the purchase by Igney and sale by Symser of an Illinois State license to Igney. According to Igney's testimony, at Smyser's suggestion the scene of these negotiations was transferred from Smyser's office to a secluded corner in the Boston Oyster House in this city, where it is testified by Igney he paid Smyser $200 in cash on account of the license which was to be issued to him, and that he had also paid Smyser other sums of money. That being in Smyser's office one day, and anxious to obtain the coveted document which would obtain for him a professional title, Smyser introduced him to a third party, one Edward Flynn, with the suggestion that Flynn would give him his license. Igney further testified, that in compliance with an appointment made by Flynn he met him at the Morrison Hotel in this city shortly afterwards, where Flynn (so Igney testifies) demanded more money, saying that there were five members of the State Board of Dental Examiners and that each of them would require $100. Igney states that he made the best deal he could, and gave up some more money and a diamond pin, the value of which the latter he estimated at about $125.

The license given Igney was No. 3030, was dated Dec. 28, 1900, and bore the names of H. W. Pitner,

A. C. Barr, W. C. Jocelyn, president, and J. H. Smyser, secretary.

The body of the blank form was admitted by Igney to have been filed out by him under Flynn's personal direction, and it recites that he had received the degree of D.D.S. from the American College of Dental Surgery of the State of Illinois, April 4, 1895, when as a matter of fact Igney had never received any such degree, had never been within the doors of that college, and had never even seen the college building.

Smyser upon the witness stand denied all knowledge and complicity in this transaction. Judge Kavanaugh, however, held him to bail to await the action of the Grand Jury.

Judge Kavanaugh in deciding the case among other things states: "Unless he (Igney) had corroboration in the testimony, I should hesitate very much to hold any one upon his testimony alone. The law requires that one sitting as an examining magistrate should be convinced that a crime has been committed, and second, that there is a reasonable ground that the person charged is guilty of the crime, but it appears without question that a crime was committed, it appears without question that Igney did get in his possession a certificate to practice to which he was not entitled. He got it from persons for whom there was no motive that could be reasonably ascribed except the motive which he gives of corruptness. Now, it may be assumed without question that he did obtain by means of bribery a certificate to practice dental surgery in this State and that one was issued to him. Therefore, I must find that a crime was committed."

On the 24th of August a further complaint was made by the chairman of the Dental Protective Association for the arrest of Flynn on a charge connected with the issuing of the Igney license. On this warrant, although Flynn has resided in this city for years, the State's officers have not been able to compass his arrest up to the time of our going to press.

Briefly told this is the sum of the work that has thus far been accomplished. What will be done in the future can not be fully estimated at this time, nor would it be wise to disclose our plans. The line of action in the matter of bogus diplomas and diploma mills is still in abeyance. In the October *Digest* we shall give in detail the testimony of the handwriting experts, showing the methods of detecting forgery, and how they arrived at their decision in this case, which will make very interesting reading. We can also promise our readers some startling disclosures next month. —Editorial in September *Digest*.

